# Emergency medical genomes: a breakthrough application of precision medicine

**DOI:** 10.1186/s13073-015-0201-z

**Published:** 2015-07-30

**Authors:** Stephen F. Kingsmore, Josh Petrikin, Laurel K. Willig, Erin Guest

**Affiliations:** Center for Pediatric Genomic Medicine, Children’s Mercy — Kansas City, Kansas City, MO 64108 USA; Department of Pediatrics, Children’s Mercy — Kansas City, Kansas City, MO 64108 USA; School of Medicine, University of Missouri — Kansas City, Kansas City, MO 64108 USA; Division of Neonatology, Children’s Mercy — Kansas City, Kansas City, MO 64108 USA; Division of Nephrology, Children’s Mercy — Kansas City, Kansas City, MO 64108 USA; Division of Hematology and Oncology, Children’s Mercy — Kansas City, Kansas City, MO 64108 USA

## Abstract

Today there exist two medical applications where relatively strong evidence exists to support the broad adoption of genome-informed precision medicine. These are the differential diagnosis of single gene diseases and genotype-based selection of patients for targeted cancer therapies. However, despite the availability of the $1000 genome and $700 exome for research, there is as yet little broad uptake of genomic medicine, even in these applications. Significant impediments to mainstream adoption exist, including unavailability in many institutions, lack of scalability in others, a dearth of physician understanding of interpreted genome or exome results or knowledge of how to translate consequent precision medicine care plans, and a lack of test reimbursement. In short, genomic medicine lacks a breakthrough application. Rapid genome sequencing of acutely ill infants with suspected genetic diseases (STATseq) may become that application when scaled to dozens of trios per day without loss of timeliness or accuracy. Also critical for broad adoption is embedding STATseq in software for timely patient ascertainment, augmented intelligence for interpretation, explanation of results for generalist physicians, and dynamic precision medicine decision support.

The Merriam-Webster dictionary defines a Killer, or breakthrough, Application as “a computer application of such great value or popularity that it assures the success of the technology with which it is associated; broadly: a feature or component that in itself makes something worth having or using” [1]. Hitherto, genomic or precision medicine has been technology-driven. In the mature genomic era, lifestyle, occupation, and healthcare choices will be bettered for the majority by genomic guidance [2]. However, in the words of Mark Kaganovich, “To be the next Internet, genomics needs its “light bulb moment” — the singularity where the technology reaches the point where applications can be built and deployed to the mainstream market leveraging the infrastructure built for and by previous applications” [3]. In the parlance of Roger’s Innovation Adoption Curve, we have entered the chasm between innovators and early adoptors [4].

For over a decade, the achievement of the $1000 genome has itself been touted as the breakthrough application — the key that enables entry into the genomic era of human self-realization, when a person’s identity becomes increasingly defined by their DNA code. Thanks to strategic investments by the National Human Genome Research Institute, as of January 2014, the $1000 genome became reality [5]. Eighteen months later, however, it is difficult to discern a consequent change in genomics other than a larger *n* (the number of subjects proposed to be studied) in genomic grant proposals, a re-ranking of the top 100 genome sequence providers, and the robust sales of HiSeq XTen systems. This lack of impact is partly the result of limitations in this version of the $1000 genome, which include scaling and turnaround times designed for large research studies (18,000 genomes per year), and the hidden cost and lack of commensurate scaling of genome analysis, interpretation, confirmatory studies, reporting, understanding and translation into precision care.

Access to a cheaper genome alone does not account for the latter, fundamental problems with the incorporation of genomic health information into medical practice. Medical genomes today are difficult to comprehend (unintelligible with byzantine resultant language), slow (typically 3–6 months), yield little actionable information relative to their potential, and most of the information comes with caveats and qualifications that few physicians understand. There is no quick solution to the paucity of deterministic or actionable information that a genome yields today in a healthy individual or in most common diseases. What will be the first breakthrough application for genomic medicine — the driver application that overcomes these barriers, persuades patients and physicians that genomes have significant health implications, and changes the genome from esoteric to the breeze block with which precision medicine is built?

## Two medical applications that are ready for genome-informed precision medicine

While the full realization of a breakthrough application for genome-guided precision medicine has yet to be recognized, there are at least two medical applications today that may serve as models for genome-informed precision medicine. These applications have a relatively high likelihood of yielding acutely actionable information. Study of these applications can inform the business and design focus to cross the adoption chasm.

The first is the differential diagnosis of single gene diseases where there has been longstanding evidence that a molecular diagnosis at or near disease onset can markedly improve outcomes. Clearly, for the approximate 60 genetic diseases tested by newborn screening (NBS) programs, this was substantiated by the implementation of a state precision medicine public health service since the late 1950s [6–8]. The NBS precision medicine program is a coordinated system of services with five parts (Box 1). The genetic diseases tested by NBS were chosen specifically based on the availability of medical therapies that when implemented immediately decreased morbidity and mortality, and prevented many, and in some disorders all, of the serious clinical sequelae. The feasibility and benefit of early diagnosis for the remaining ~4300 genetic diseases has started to be addressed in six recent retrospective case series. Totaling 3587 subjects, these studies reported molecular diagnostic yields of 27–57 % (Table 1) [9–13]. Furthermore, two of these reported that diagnoses changed acute clinical management in 49–100 % of patients, findings which start to overcome the general misconception that nothing can be done for most genetic diseases (Table 1). While no prospective studies of consequent change in outcomes have yet been published, the retrospective evidence is strengthened by an abundance of case reports of the clinical utility of genome- or exome-derived diagnoses.

**Table 1:** Results of five large, retrospective case studies of the diagnostic rate of genome or exome sequencing in children with suspected genetic diseases, particularly neurodevelopmental disabilities

Reference	Number of subjects	Disease	Age in years (mean or median)	Diagnosis rate	De novo mutation	Management changed by diagnosis
[9]	100	NDD	7	47 %	51 %	49 %
[10]	78	NDD	9	41 %	56 %	100 %
[11]	1756	Any	6	27 %	49 %	Not examined
[12]	520	Any	<18	26 %	50 %	Not examined
[13]	1133	NDD	6	27 %	62 %	Not examined

The second medical application where genome sequences have a relatively high likelihood of yielding acutely actionable information today is in oncology. The landscape of cancer genomics is rapidly being described through efforts of large collaborative groups, including The Cancer Genome Atlas (TCGA) of the National Cancer Institute (NCI), the International Cancer Genome Consortium (ICGC), and the Pediatric Cancer Genome Project [14–16]. Genomic biomarkers have the potential to aid with cancer diagnosis and classification, prognosis and, most importantly, molecularly guided treatment [17]. While the diagnosis and treatment of cancer has historically been based upon histologic findings and extent of disease, cancers are now being reclassified by molecular subtype, with treatment tailored to the pathways mutated. For example, recurrent and potentially targetable genetic alterations that are predictive of poor outcome have been described in childhood acute lymphoblastic leukemia (ALL) [18, 19]. Genotype-based selection of patients for the application of targeted therapies has already had a substantial impact on the treatment of some cancers, such as tyrosine kinase inhibitors in patients with nonsmall cell lung cancers [20–24]. Furthermore, precision oncology represents a specialized case of pharmacogenomics, where genome information can guide both the choice of drug and the drug exposure, based on ADME (absorption, distribution, metabolism, and excretion) variants.

Prospective trials of the tumor genome, exome, and gene panel-guided treatments are now in progress. For example, the Lung Cancer Master Protocol (Lung-MAP) trial is examining whether targeted cancer therapy cocktails that are matched to the genomic makeup of the squamous cell lung cancer tumors of patients are more effective than the current standard therapy in halting or reversing the progress of the disease and in extending the life of the patient [25]. Other such studies in development are the NCI-Molecular Profiling-Based Assignment of Cancer Therapy for Patients With Advanced Solid Tumors (NCI-MPACT; ClinicalTrials.gov Identifier: NCT01827384), NCI-Molecular Analysis for Therapy Choice (NCI-MATCH), and Pediatric MATCH [26, 27]. These prospective trials remain limited to patients who have exhausted standard treatment options and who have relapsed and/or have refractory cancer. Despite a greater understanding of signaling pathways, tumor heterogeneity, clonal evolution, treatment resistance, and the importance of epigenomic alterations, precision oncology is in its infancy [27–31]. The results of clinical trials incorporating comprehensive genomics data will help describe the role of next-generation sequencing in cancer diagnostics and therapeutics [32, 33].

However, neither of these clinical applications has yet risen to the level of the genomic breakthrough application. Physicians generally do not yet practice precision medicine in such clinical situations. Lack of physician familiarity with the interpretation of genome or exome tests, or of the guidelines for changes in management following genomic test results, undoubtedly explains part of a slow uptake of physician-ordered testing. Additionally, in the current era of evidence-based, standardized management protocols, the use of precision medicine, focused on individualized care plans, is counterintuitive. Refusal of payors to reimburse clinical genomes and exomes is also a great hindrance to broad utilization. However, a less frequently considered issue is the lack of scalable, timely results. The turnaround time for results from a medical genome or exome is typically 6 weeks to 6 months, making the medical genome possibly the most cumbersome diagnostic test in the world.

## Speed heals

A key ingredient for the breakthrough application of genomic medicine is speed at scale. In medical practice, the value of information is proportionate to its immediacy relative to the acuity of the clinical situation. Today, medical genomes — even in the two applications for which there exists clear evidence of utility — rank as 'last resort' tests in diagnostic workups because results will not be available at that clinic visit, or during that on-service period, or during that hospitalization. Genomes will only become an integral part of inpatient rounds, surgical pre-ops, admission orders, newborn panels, and many outpatient clinics in these two application areas when the time-to-result routinely matches the acuity of the clinical situation. Consequently, we think that rapid genome sequencing — STATseq — embedded within precision medicine software programs will be the first breakthrough application of medical genomics. The STAT in STATseq comes from the Latin word “statim”, which means immediately. In medical parlance, a blood test that is ordered STAT is one that needs to be performed as an emergency. The seq in STATseq is an abbreviation for next-generation sequencing, as in “RNAseq” for RNA sequencing. When we were children, Star Trek was a popular television and film series. The tricorder was a portable sensing, computing and recording device used by Star Trek doctors to help diagnose diseases and collect biomarker data about patients. While, ultimately, we desire the genomic equivalent of the Star Trek medical tricorder, shortening the scalable turnaround of STATseq to 24 h using software to guide physician understanding and provision of precision medicine is needed to catapult medical genomes into the consciousness of physicians.

In 2012, we published a proof of concept paper for STATseq, in which we showed the feasibility of diagnosing genetic diseases in 50 h through whole genome sequencing [34]. Of two retrospective cases and four prospective cases, STATseq yielded a molecular diagnosis in five. There were two material developments that contributed to the 50-h medical genome. First was the availability of a sequencing instrument that could generate over 120 GB of DNA sequence in 26 h (the HiSeq 2500 in rapid-run mode). Hitherto, the only high output run modes that were available took 11 days. The second was the implementation of informatics processes that largely automated the search for a diagnosis. Specifically, the clinical features of an individual patient (the phenome) were entered, automatically mapped to the canonical clinical features of all known genetic diseases, and ordered by the goodness of fit. The genes corresponding to the genetic disease hits were then orthogonally overlaid on all genomic variants. When performed together with variant filtering on the basis of rare occurrence in populations, inheritance models, and the evidence of being pathogenic, this approach frequently can yield a singular diagnosis. In principle, this process could be automated, with ascertainment of clinical features from the electronic medical record, derivation of a comprehensive genetic differential diagnosis, and orthogonal analysis of filtered genomic variants. Akin to an autopilot, we envisage augmented intelligence systems supervised by diagnostic laboratory directors and clinicians.

**Fig. 1:**
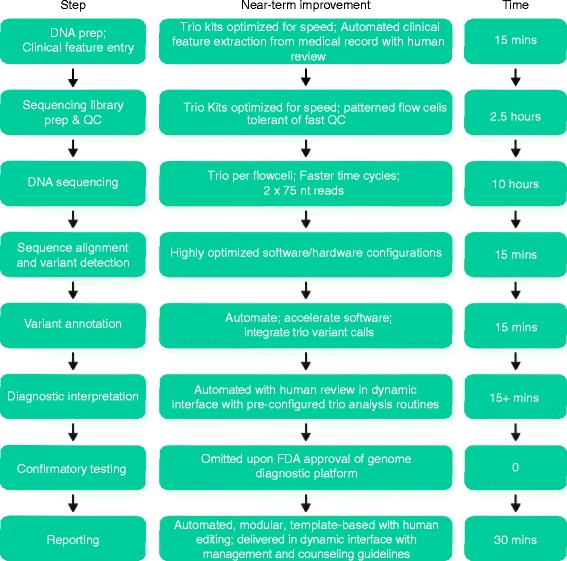
Near-term improvements in clinical genomes to enable 14 h time to molecular diagnosis of genetic disease. Note that time of interpretation us highly variable. Fifteen minutes is a lowest estimate. Abbreviations: *FDA* US Food and Drug Administration; *nt* nucleotide, *QC* quality control

What are the other practical steps that will reduce STATseq from 50 h to 18 h and scale from one trio per week to dozens per day (Fig. 1)? There are several options, and the good news is that 18 h is within reach in the next 2 years without any transformative, unforeseen, novel technologies. Firstly, faster sequencing library preparation and an ultra-rapid run mode are feasible for the Illumina HiSeq platform [35, 36]. Faster cycle times enable 2 × 101 cycles to be performed in 18 h, rather than the standard 26 h, without loss of sequence quality or cluster density [36]. Secondly, as Stranneheim et al. [37] have shown, shorter read lengths permit faster time-to-results. They described pulsed whole genome sequencing with analysis of results iteratively at 35, 50, 75 and 100 cycles. There is minimal loss of sensitivity or specificity with 2 × 75 cycle sequences when compared with 2 × 101 cycles using current library preparation methods and alignment and variant calling algorithms. With a patterned flow cell, it is possible to increase the cluster density so that 2 × 75 cycle sequencing generates sufficient genomic coverage to retain high sensitivity in a trio. Thirdly, genome scale, highly sensitive alignment, variant calling, and annotation are now possible in less than 1 h. Examples of such algorithms and hardware are iSAAC and DRAGEN [38, 39]. Finally, rapid exome enrichment methods are now available that largely circumvent the need for costly whole genome sequencing to achieve 2-day turnaround times.

## Emergency genome sequencing in newborn intensive care units

The second ingredient for the genome breakthrough application is the ability to automate patient ascertainment, diagnosis, medical translation, and precision clinical management through the use of software and artificial (augmented) intelligence. The first medical application examined for the clinical utility STATseq was genetic disease diagnosis in acutely ill infants [34]. This population was selected based on the high prevalence of suspected genetic disease, the anticipated impact on clinical management, the perceived need for a more rapid molecular testing mechanism due to patient acuity, and the high healthcare costs for this population. Clearly, there exists an immense unmet medical need in these patients; genetic diseases and congenital anomalies are the leading cause of death in infants [40]. Such infants typically are cared for in level III–IV neonatal intensive care units (NICU) or pediatric intensive care units (PICUs), facilitating automated ascertainment, and underscoring the need for short turnaround times not provided by current clinical testing. From a cost-effectiveness standpoint this is also a unique medical application given that costs average $73,000 for a level II–IV NICU stay and there is potential for more than 70 added quality-adjusted life years (QALYs) by timely identification of a treatable condition in an individual infant [41].

Our recent report of initial retrospective experience with STATseq in this application is informative for understanding the practical bottlenecks in implementation of genomic medicine in this population [42]. Thirty-five infants aged <4 months were enrolled from the NICU and PICU of our children’s hospital who had an acute illness of suspected genetic etiology. Despite a goal of recruitment at birth, in practice the average age at enrollment for STATseq was day of life 26. Despite the potential for a 50-h time to result, in practice the median time to genome analysis was 5 days, and 23 days to Sanger-confirmed STATseq diagnostic report [42]. While these times were skewed somewhat due to factors such as lack of familiarity with STATseq, newly discovered disease genes and ongoing methods improvements, they illustrate the current lack of scalability of STATseq, and need for the technical innovations noted above.

STATseq was very effective for diagnosis of genetic diseases in these infants. The rate of diagnosis of a genetic disease was 57 % by STATseq and 9 % by conventional diagnostic testing; 65 % of STATseq diagnoses were associated with de novo mutations, underscoring the need for simultaneous STATseq of trios (parents and their affected infant) [42]. Mortality at day of life 100 was 57 % among infants receiving a genetic diagnosis. Thus, the interval between the return of results and death was extremely brief, allowing very limited opportunity for consideration of precision, nonstandard treatments. Nevertheless, genetic disease diagnoses frequently had an impact on medical management. In infants receiving a STATseq diagnosis, acute clinical utility was observed in 65 %. A strongly favorable impact on management occurred in 20 % of diagnoses, and palliative care was instituted in 30 %.

This study showed that, while STATseq is effective for genetic disease diagnosis in acutely ill infants, patient ascertainment/enrollment could be considerably earlier (for example, on the first NICU day), STATseq trio time-to-result must be fast at scale, and return of results should be in the setting of infrastructure for immediate consideration and implementation of precision medicine, in order to maximize the potential for improved outcomes. A template for the latter exists; for each genetic disorder tested by newborn screening programs, the American College of Medical Genetics has developed: 1) an ACTion (ACT) sheet that describes the short-term actions a health professional should follow in communicating with the family and determining the appropriate steps in the follow-up of the infant that has screened positive; and 2) an algorithm that presents an overview of the basic steps involved in determining the final diagnosis in the infant. This is accompanied by specific management guidelines for each specific disease that cover rapid planning and implementation of long-term therapy (Box 1) [43].

Given the heterogeneity of genetic diseases, a key next step for precision NICU medicine is to combine neonatal genetic diseases into groups for which common, structured sets of precision interventions can be developed and implemented at scale. Based on preliminary insights from our retrospective case series and case reports [42, 44, 45], we would then envisage return of results of STATseq together with structured, precision medicine treatment algorithms. These could either be based on common complications of NICU genetic disease, such as seizures, hypoglycemia, metabolic acidosis, or cardiac dysrhythmias (akin to clinical trial designs of LungMAP, NCI-MATCH and NCI-MPACT in precision oncology), or based on common molecular pathways of disease. These would include, for example, ACT sheets for available protein therapeutics and medical diets. Where the prognosis is hopeless, a precision palliative care plan could be elaborated that is calibrated on minimization of infant suffering and best practices for support of grieving parents and siblings.

## Reimbursement

Historically, payors in the United States have resisted reimbursement of genetic tests. In part this was because the individual tests for 4500 disease genes were infrequent, the CLIA/CAP licensed laboratories where testing was predominantly undertaken were small and widely disbursed, and therefore the lobby for reimbursement was fragmented and lacked the clout of other clinical specialty societies. Now that genetic testing is being consolidated into genomes, exomes and specific panels, and amongst fewer laboratories, there is the opportunity, for the first time, for a unified lobby for reimbursement of diagnostic testing for rare genetic diseases. Ongoing efforts to reduce the total cost of clinical genome testing and to improve the range of types of mutations that are detected will be important in reimbursement. Also critical, however, will be prospective, randomized clinical studies that not only address diagnostic yield of genome sequencing, but also the clinical utility and cost effectiveness of consequent provision of precision medicine.

## Conclusions

A comprehensive system for delivery of acute precision care is anticipated to be the first breakthrough application for genomics in areas such as neonatology and oncology. As has occurred for other technologies, the breakthrough application will drive the development of additional genomics infrastructure investments. These will include genome-capable electronic medical records, regional genome sequencing capability across the US in hospitals, and reference laboratories. Likewise, the first breakthrough application will drive physician and patient familiarity and acceptance, which will facilitate a second generation of applications in segments such as pediatric endocrinology, pediatric neurology, general oncology, and broader applications of pharmacogenomics.

## Box 1. The five components of newborn screening precision medicine of selected genetic diseases (from [43])

1. Screening: Heel-prick testing of newborns at about 24 h of age. Blood spots (Guthrie cards) are sent to a state newborn screening lab. Results are returned to the newborn’s physician within 10–14 days.
2. Follow-up: Rapid location, follow-up, and referral of infants with positive (abnormal) screening test results.
3. Diagnosis: Structured evaluations of infants with a positive screening test to make a definitive diagnosis or exclude the disorder.
4. Management: Rapid planning and implementation of long-term therapy. Specific management guidelines exist for each specific disease, and include the required expertise of healthcare providers, parental health education, health maintenance, management of acute illness, genetic counseling, and psychosocial support.
5. Evaluation: Validation of testing procedures, assessment of the efficiency of follow-up and intervention, and assessment of the benefit to the patient, family, and society.

## Abbreviations

ACT: ACTion [sheet]
ALL: Acute lymphoblastic leukemia
ICGC: International Cancer Genome Consortium
Lung-MAP: Lung Cancer Master Protocol
NBS: Newborn screening
NCI: National Cancer Institute
NCI-MATCH: National Cancer Institute-Molecular Analysis for Therapy Choice
NCI-MPACT: National Cancer Institute-Molecular Profiling-Based Assignment of Cancer Therapy for Patients With Advanced Solid Tumors
NICU: Neonatal intensive care unit
PICU: Pediatric intensive care unit
QALY: Quality-adjusted life year
STATseq: Rapid medical whole genome sequencing
TCGA: The Cancer Genome Atlas
